# Determination of Activity Concentration of Natural Radionuclides and Radiation Hazards' Assessment of Building Materials in High Background Radiation Areas of Homa and Ruri, Kenya

**DOI:** 10.1155/2021/9978619

**Published:** 2021-12-14

**Authors:** Willis Otieno Gor Odongo, Margaret Chege, Nadir Hashim, Shinji Tokonami, Kranrod Chutima, Charles Rotich

**Affiliations:** ^1^Department of Physics, Kenyatta University, P.O. Box 43844, Nairobi, Kenya; ^2^Department of Physics, Kisii University, P.O. Box 408, Kisii, Kenya; ^3^Institute of Radiation Emergency Medicine, Hirosaki University, Hirosaki, Aomori, Japan

## Abstract

The areas around Homa and Ruri hills in Homa Bay County in Kenya are associated with high background radiation levels. The activity concentration of the natural radionuclides (^226^Ra, ^232^Th, and ^40^K) in earthen building materials used in the areas of Homa and Ruri hills has been measured using a NaI (Tl) detector in this work. The measured values of radioactivity concentrations are used to estimate the associated radiological risk. The earthen building material samples from Ruri registered relatively high ^232^Th concentration values averaging 1094 ± 55 Bq/kg, nearly three times those of the samples from Homa. ^226^Ra level was not significantly different in both regions with Homa reporting 129 ± 10 Bq/kg and Ruri 111 ± 6 Bq/kg. ^40^K was however higher in the samples from Homa by an approximate factor of 2 relative to those from Ruri where the activity concentration was 489 ± 24 Bq/kg. The radium equivalents for ^226^Ra, ^232^Th, and ^40^K in the samples from Ruri were 111 ± 9, 1564 ± 125, and 38 ± 3 Bq/kg, while in Homa, the values were 129 ± 10, 570 ± 46, and 69 ± 5 Bq/kg, respectively. The calculated value of total radium equivalent in Ruri was 1713 ± 137 Bq/kg which was two times higher than that of Homa. ^232^Th contributed about 74% and 91% to the total radium equivalent in Homa and Ruri, respectively; thus, it was the one with the largest contribution to radiation exposure in both regions. The average indoor annual effective dose rates were 1.74 ± 0.14 and 3.78 ± 0.30 mSv/y in Homa and Ruri, respectively, both of which were above the recommended safety limit of 1 mSv/y.

## 1. Introduction

Natural radiation in the environment contributes approximately eighty percent of the total radiation exposure to the general public. The major categories of natural exposure include inhalation of radon and thoron, external exposure from (^226^Ra, ^232^Th, and ^40^K), cosmic radiation, and ingestion of food and water [1]. The major natural contributors to external exposure are the primordial radionuclides ^226^Ra, ^232^Th, and ^40^K which are not uniformly distributed in the environment but occur in varying quantities in rock and soil as characterized by the geology of a region [2]. The average worldwide dose rate as a result of these terrestrial radionuclides is about 60 nGy^−1^ for areas with normal background. Therefore, it is important to determine their levels in soil and rocks, as well as their individual contributions to the total radiation dose for purposes of radiation protection and management [3]. High background radiation areas (HBRAs) are characterized by abnormally high levels of background radiation; they are distributed throughout the world, e.g., Yangjiang, China; Guarapari, Brazil; Ramsar, Iran; and Kerala, India [4, 5, 6, 7]. In Kenya, some of the HBRAs include Mrima hill in the coastal part of Kenya and Homa and Ruri in southwestern Kenya [8, 9, 10]. Studies carried out, for instance, in high background radiation areas of Ramsar, Iran [11], and Mrima hill, Kenya, have shown that building materials contribute significantly to indoor radiation exposure. Homa and Ruri are both experiencing increased growth in human settlement with readily available soil being used as a building material. Therefore, there exist a potential radiation risk indoors from the soil used as the building material in these regions and the fact that people generally spend more time indoors. Despite this apparent risk, there are no data on radiation exposure as a result of the terrestrial radionuclides inside the local earthen dwellings in Homa and Ruri which this research seeks to determine. This paper reports the activity concentration of the primordial radionuclides in the earthen building materials used in the two regions as well as the risk indices associated with them. The radionuclides responsible for the highest radiation exposure are also determined.

## 2. Methodology

### 2.1. Study Area

Homa and Ruri hills are located in Homa Bay County along the shores of Lake Victoria in Kenya.

Homa hill is located between latitude 0° 30′ N and 0^o^ 20′ N and longitude 33^o^ 26′ E and 34^o^ 34′ E. This is mainly covered by a large carbonatite peninsula complex on the eastern shores of Lake Victoria with a series of cone sheets of carbonatite and breccia intrusions in the oldest rock in the Nyanzian series and ijolites [12].

**Figure 1:**
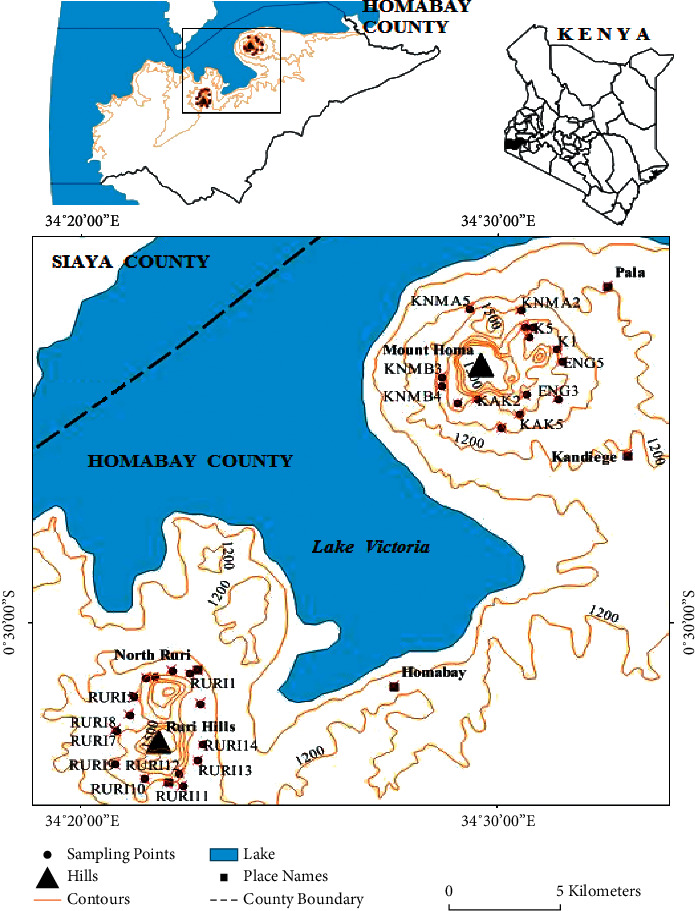
A map of Homa Bay County showing both Homa and Ruri hills (sample points marked with the dots around each hill).

Ruri hill is located at latitudes 0^o^ 30′ S and 1^o^ 00′ S and longitude 34^o^ 30′ E and Lake Victoria shoreline. The altitudes range from about 1000 m to approximately 1800 m at the hilltop. This area is mainly covered by Precambrian metabasalt of the Nyanzian type of rocks composed of ijolites and the nepheline syenites [13]. The hill also has a ring-shaped intrusion of carbonatites of lower tertiary age and monazite and pyrochlore minerals associated with high ^232^Th levels [14].  Figure 1 shows the map of Homa Bay County and the two hills with the sampling points marked with the dots around each region.

### 2.2. Sample Collection and Its Preparation

Fifteen soil lumps were chopped off from the earthen walls of randomly selected houses in each region. In the laboratory, each lump was crushed to a fine powder before drying in an oven at a temperature of 110°C for a period of 24 hours. 250 g mass of each soil sample was then sealed in a Marinelli beaker, labelled, and stored for about 4 weeks to attain radioactive secular equilibrium between ^226^Ra (^238^U decay chain) series and ^232^Th series and their daughters [15].

## 3. Experimental Techniques

### 3.1. Radionuclide Concentration Analysis Using Gamma-Ray Spectroscopy (NaI (Tl))

The gamma-ray spectrometer used in this work is composed of a 76 mm × 76 mm thallium-activated sodium iodide (NaI (Tl)) single-crystal detector and an Oxford PCA-P multichannel analyzer which is a PC-based plugin PCI card. It consists of an 80 MHz Wilkinson analogue-to-digital converter for spectral data acquisition. The energy calibration of the detector was done using caesium-137 at the energy peak of 662 keV and cobalt-60 at energy peaks of 1170 keV and 1330 keV. The detector efficiency calibration was done using International Atomic Energy Agency (IAEA) standard-certified reference materials RGU-1, RGTh-1, and RGK-1 having the same geometry as the samples, and each was counted for a period of 30,000 seconds. ^226^Ra and ^232^Th activity concentrations were determined based on the ^214^Bi gamma energy peak of 609 keV and ^208^Tl at the energy peak of 2615 keV, respectively, while for ^40^K, an energy peak of 1460 keV was used from the spectrum of the background counting. The background counts were then used for the correction of net peak area of gamma rays of the measured standard isotopes. The minimum detectable activity (MDA) for ^40^K, ^232^Th, and ^226^Ra was determined as 1.4, 0.196, and 0.401 Bq, respectively.

The activity concentration was determined by using the following equation [15]:

(1) $$\begin{array}{c} A_{i}=\frac{N}{\gamma mnt}, \end{array}$$

where *A*_*i*_ is the activity concentration of radionuclide *i*, *N* is the residual net counts at the peak energy of interest, *γ* is the emission probability of the gamma ray of interest, *m* is the mass of the sample in kg, *n* is the detection efficiency of the gamma ray of interest, and *t* is the acquisition time in seconds.

### 3.2. Radium Equivalent Activity (Ra_eq_)

Radium equivalent activity is a single value that describes the gamma output from the terrestrial natural radionuclides as determined by the following equation [15]:

(2) $$\begin{array}{c} Ra_{\mathrm{eq}}=A_{\mathrm{Ra}}+1.429A_{\mathrm{Th}}+0.0769A_{\text{K}}, \end{array}$$

where *A*_Ra_, *A*_Th_, and *A*_K_ are the activity concentrations of ^226^Ra, ^232^Th, and ^40^K, respectively. 1.429 and 0.0769 are conversion factors for ^232^Th and ^40^K, respectively.

### 3.3. Absorbed Gamma Radiation Dose Rate (*D*)

Absorbed gamma radiation dose rate is the dose of ionizing radiation per unit time and is dependent on the concentration of the terrestrial radionuclides in the earthen building materials. The absorbed gamma dose rate *D* (nGy/h) in air considered 1 m above the ground surface was determined using the following equation [1]:

(3) $$\begin{array}{c} D=0.462A_{\mathrm{Ra}}+0.604A_{\mathrm{Th}}+0.0417A_{\text{K}}, \end{array}$$

where 0.462, 0.604, and 0.0417 nGyh^−1^/Bqkg^−1^ are dose conversion factors for ^226^Ra, ^232^Th, and ^40^K, respectively, and *A*_Ra_, *A*_Th_, and *A*_K_ are the activity concentrations of ^226^Ra, ^232^Th, and ^40^K in Bq/kg, respectively.

### 3.4. Indoor Annual Effective Absorbed Dose Rate (AEDR)

Indoor annual effective absorbed dose rate is the measure of biological effect of radiation on humans inside a dwelling made of the soil. It was determined by the following equation [1, 16, 17]:

(4) $$\begin{array}{c} \text{AEDR}=D\left(\frac{nGy}{h}\right)\times 8,760h\times 0.6\times 0.7\left(\frac{Sv}{Gy}\right), \end{array}$$

where AEDR is the indoor annual effective absorbed dose rate in mSv/y, *D* is the absorbed dose rate in nGy/h, 8760 is the time in hours for a whole normal year of 365 days, 0.6 is the rural Kenya indoor occupancy factor [8], and 0.7 Sv/Gy is the gamma dose conversion factor; 1.4 is a factor that accounts for the indoor environment given that gamma dose rates indoor are about 1.4 times higher than outdoors [8, 16, 17].

## 4. Results and Discussion

The activity concentration of ^226^Ra, ^232^Th, and ^40^K in Homa and Ruri is summarized in Tables 1 and 2, respectively. In both regions, the activity concentration of ^226^Ra had no significant difference between them with average values of 129 ± 10 Bq/kg and 111 ± 6 Bq/kg. respectively. The average value of ^232^Th was approximately 60% higher in Ruri compared to Homa which had an average of 399 ± 20 Bq/kg; this was attributed to the ring intrusion of monazite and pyrochlore minerals in Ruri associated with higher thorium concentration [13]. On the contrary, the average activity concentration of ^40^K was about 40% higher in Homa than Ruri which had an average of 489 ± 24 Bq/kg, which was attributed to the alkaline igneous rocks in Homa associated with higher potassium levels [18]. Average activity concentration of ^226^Ra was approximately 3 times higher than the world average of 35 Bq/kg in both Homa and Ruri.

The average activity concentration of ^232^Th was 13 and 36 times higher than the world average of 30 Bq/kg in Homa and Ruri, respectively. The arithmetic mean of ^40^K was twice that of the world average of 400 Bq/kg in Homa but was nearly equal to the mean value in Ruri [1]. Radium equivalent (Ra_eq_) for ^226^Ra, ^232^Th, and ^40^K and the total radium equivalent in Homa and Ruri are presented in Tables 3 and 4, respectively. The average radium equivalents for ^226^Ra were more or less the same in both regions given their nearly equal activity concentrations. Ra_eq_ for ^232^Th was 60% higher in Ruri which had an average of 1564 ± 125 Bq/kg, while ^40^K in Homa was higher by a factor of 2 relative to Ruri which was 38 ± 3 Bq/kg.

**Figure 2:**
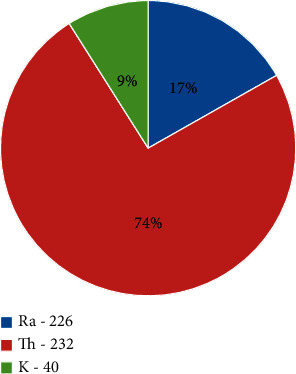
Percentage contributions of ^226^Ra, ^232^Th, and ^40^K to total Ra_eq_ in the 15 samples from Homa area analyzed in this work.

**Figure 3:**
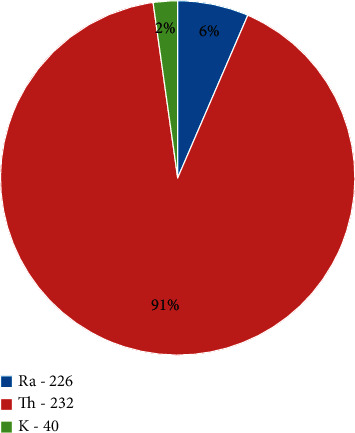
Percentage contributions of ^226^Ra, ^232^Th, and ^40^K to total Ra_eq_ in the 15 samples from Ruri area analyzed in this work.

Figures 2 and 3 show pie chart representation of the contribution of ^226^Ra, ^232^Th, and ^40^K to total Ra_eq_ in Homa and Ruri, respectively. In Homa hill, ^232^Th contributed 74% as ^40^K contributed 9% to total Ra_eq_ despite ^40^K having the highest activity concentration, while in Ruri, ^232^Th contributed 91% to total Ra_eq_, the lowest contributor still being ^40^K at just 2% of total Ra_eq_. ^232^Th was therefore the highest contributor to the total radium equivalent and radiation exposure in both regions. The determined average total radium equivalent in Homa was 767 ± 61 Bq/kg which was just 40% of the total average radium equivalent in Ruri.

**Figure 4:**
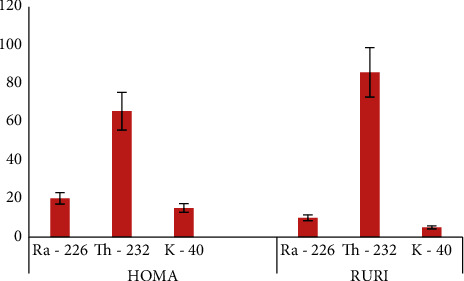
Percentage contribution of ^226^Ra, ^232^Th, and ^40^K to the indoor annual effective dose rate.

The indoor annual effective dose rate (*D*) is determined from the absorbed gamma radiation dose rates in Tables 3 and 4 for Homa and Ruri, respectively, using equation (4). The average annual effective dose rate in Homa and Ruri was 338 ± 30 nGy/h and 733 ± 66 nGy/h, respectively, both of which were above the world average of 84 nGy/h [19]. The average annual effective dose rate in Homa was 1.74 ± 0.14 mSv/y which was about half that of Ruri.  Figure 4 shows a bar graph presentation of the percentage contribution of ^226^Ra, ^232^Th, and ^40^K to the total annual effective dose rate. ^232^Th contributed the highest percentage of about 65% and 85% to the indoor annual effective dose in Homa and Ruri, respectively, compared to ^40^K and ^226^Ra. ^40^K contributed the least to the effective dose in both regions despite it having a high activity concentration. Approximately 80% of the sampled points in Homa had indoor AEDR above the recommended safety limit of 1 mSv/y, while all the sampled points in Ruri were above this limit [19].

The results obtained in this work have been compared with results reported in building materials in other high background radiation areas in Kenya and others around the world as tabulated in Table 5 [8, 11, 20, 21]. ^40^K was 70% and 50% higher in Homa and Ruri, respectively, compared to Mrima hill, Kenya. On the contrary, ^232^Th was 60% higher in Ruri compared to Mrima which attributed to monazite and pyrochlore minerals in Ruri which contains higher thorium levels [1].

## 5. Conclusion

The levels of activity concentration of ^226^Ra, ^232^Th, and ^40^K in earthen building materials used in high background radiation areas of Homa and Ruri have been assessed using the NaI (Tl) detector. The average concentration of ^226^Ra, ^232^Th, and ^40^K was above the world average values of 35 Bq/kg, 30 Bq/kg, and 400 Bq/kg, respectively [1]. The radium equivalent, absorbed gamma radiation dose rate, and indoor annual effective dose rate have also been determined from the measured activity concentrations. ^232^Th was the highest contributor to the total radium equivalent and indoor annual effective dose rates in both Homa and Ruri; it is therefore the radionuclide responsible for the largest radiation exposure in the two regions attributed to high monazite levels associated with high ^232^Th levels. The determined average indoor annual effective dose rates were all above the recommended safety limit of 1 mSv/y in both Homa and Ruri [19]. Therefore, the earthen building materials in both hills are not safe for the construction of the dwellings.

## Figures and Tables

**Table 1:** Activity concentration of ^40^K, ^226^Ra, and ^232^Th (Bq/kg) in Homa samples.

Sample ID	^226^Ra (Bq/kg)	^232^Th (Bq/kg)	^40^K (Bq/kg)
Homa1	259 ± 21	598 ± 30	946 ± 47
Homa2	178 ± 14	670 ± 34	1294 ± 65
Homa3	67 ± 5	493 ± 25	1036 ± 52
Homa4	129 ± 10	631 ± 32	1103 ± 55
Homa5	31 ± 2	119 ± 6	612 ± 31
Homa6	216 ± 17	353 ± 18	766 ± 38
Homa7	51 ± 4	331 ± 17	753 ± 38
Homa8	45 ± 4	513 ± 26	1033 ± 52
Homa9	157 ± 13	126 ± 6	789 ± 39
Homa10	16 ± 2	84 ± 4	550 ± 28
Homa11	223 ± 18	420 ± 21	991 ± 50
Homa12	220 ± 18	417 ± 21	827 ± 41
Homa13	78 ± 6	378 ± 19	964 ± 48
Homa14	191 ± 15	479 ± 24	850 ± 43
Homa15	72 ± 6	367 ± 18	889 ± 44
Average	129 ± 10	399 ± 20	894 ± 45

**Table 2:** Activity concentration of^40^K, ^226^Ra, and ^232^Th (Bq/kg) in Ruri samples.

Sample ID	^226^Ra (Bq/kg)	^232^Th (Bq/kg)	^40^K (Bq/kg)
Ruri1	92 ± 5	1190 ± 60	580 ± 29
Ruri2	103 ± 5	1843 ± 92	451 ± 23
Ruri3	81 ± 4	724 ± 36	526 ± 26
Ruri4	21 ± 2	403 ± 20	333 ± 17
Ruri5	63 ± 3	2152 ± 108	320 ± 16
Ruri6	71 ± 4	1550 ± 78	742 ± 37
Ruri7	110 ± 6	1486 ± 74	739 ± 37
Ruri8	226 ± 11	1058 ± 53	487 ± 24
Ruri9	145 ± 7	896 ± 45	566 ± 28
Ruri10	189 ± 9	929 ± 46	306 ± 15
Ruri11	46 ± 2	873 ± 44	156 ± 8
Ruri12	87 ± 4	1236 ± 62	731 ± 37
Ruri13	196 ± 10	1201 ± 60	812 ± 41
Ruri14	174 ± 9	580 ± 29	358 ± 18
Ruri15	68 ± 3	298 ± 15	221 ± 11
Average	111 ± 6	1094 ± 55	489 ± 24

**Table 3:** Radium equivalent (Ra_eq_), absorbed gamma radiation dose rate (*D*), and indoor annual effective dose rate (AEDR) in Homa.

Sample ID	^226^Ra Ra_eq_ (*A*_Ra_)	^232^Th Ra_eq_ (1.429A_Th_)	^40^K Ra_eq_ (0.0769*A*_k_)	Ra_eq_ (total) (Bg/kg)	*D* (nGy/h)	Indoor AEDR (mSv/y)
Homa1	259 ± 21	855 ± 68	73 ± 6	1186 ± 95	520 ± 47	2.68 ± 0.21
Homa2	178 ± 14	957 ± 80	100 ± 8	1235 ± 99	541 ± 49	2.79 ± 0.22
Homa3	67 ± 5	704 ± 63	80 ± 6	851 ± 68	372 ± 33	1.92 ± 0.15
Homa4	129 ± 10	902 ± 71	85 ± 7	1116 ± 89	487 ± 44	2.51 ± 0.20
Homa5	31 ± 2	170 ± 49	47 ± 4	248 ± 20	112 ± 10	0.58 ± 0.05
Homa6	216 ± 17	504 ± 53	59 ± 5	779 ± 62	345 ± 31	1.78 ± 0.14
Homa7	51 ± 4	473 ± 51	58 ± 5	582 ± 47	255 ± 23	1.31 ± 0.11
Homa8	45 ± 4	733 ± 63	79 ± 6	858 ± 69	374 ± 34	1.93 ± 0.15
Homa9	157 ± 13	180 ± 56	61 ± 5	398 ± 32	182 ± 16	0.94 ± 0.07
Homa10	16 ± 1	120 ± 3	42 ± 3	178 ± 14	81 ± 7	0.42 ± 0.03
Homa11	223 ± 18	600 ± 60	76 ± 6	899 ± 72	398 ± 36	2.05 ± 0.16
Homa12	220 ± 18	596 ± 57	64 ± 5	879 ± 70	388 ± 35	2.00 ± 0.16
Homa13	78 ± 6	540 ± 68	74 ± 6	692 ± 55	305 ± 27	1.57 ± 0.13
Homa14	191 ± 15	684 ± 59	65 ± 5	941 ± 75	413 ± 37	2.13 ± 0.17
Homa15	72 ± 6	524 ± 57	68 ± 5	665 ± 53	292 ± 26	1.50 ± 0.12
Average	129 ± 10	570 ± 46	69 ± 5	767 ± 61	338 ± 30	1.74 ± 0.14

**Table 4:** Radium equivalent (Ra_eq_), absorbed gamma radiation dose rate (*D*), and indoor annual effective dose rate (AEDR) in Ruri.

Sample ID	^226^Ra Ra_eq_ (*A*_Ra_)	^232^Th Ra_eq_ (1.429A_Th_)	^40^K Ra_eq_ (0.0769*A*_k_)	Ra_eq_(Total) (Bg/kg)	*D* (nGy/h)	Indoor AEDR (mSv/y)
Ruri1	92 ± 7	1701 ± 136	45 ± 4	1837 ± 147	221 ± 20	1.14 ± 0.09
Ruri2	103 ± 8	2634 ± 211	35 ± 3	2771 ± 222	267 ± 24	1.38 ± 0.11
Ruri3	81 ± 6	1035 ± 83	40 ± 3	1156 ± 92	446 ± 40	2.30 ± 0.18
Ruri4	21 ± 2	576 ± 46	26 ± 2	622 ± 50	497 ± 45	2.56 ± 0.20
Ruri5	63 ± 5	3075 ± 246	25 ± 2	3163 ± 253	555 ± 50	2.86 ± 0.23
Ruri6	71 ± 6	2215 ± 177	57 ± 5	2343 ± 187	632 ± 57	3.26 ± 0.26
Ruri7	110 ± 9	2123 ± 170	57 ± 5	2290 ± 183	661 ± 60	3.41 ± 0.27
Ruri8	226 ± 18	1512 ± 121	37 ± 3	1775 ± 142	764 ± 69	3.94 ± 0.31
Ruri9	145 ± 12	1280 ± 102	44 ± 3	1469 ± 118	785 ± 71	4.05 ± 0.32
Ruri10	189 ± 15	1328 ± 106	24 ± 2	1540 ± 123	817 ± 74	4.21 ± 0.34
Ruri11	46 ± 4	1248 ± 100	12 ± 1	1306 ± 104	850 ± 76	4.38 ± 0.35
Ruri12	87 ± 7	1766 ± 141	56 ± 4	1909 ± 153	979 ± 88	5.05 ± 0.40
Ruri13	196 ± 16	1716 ± 137	62 ± 5	1975 ± 158	1000 ± 90	5.15 ± 0.41
Ruri14	174 ± 14	829 ± 66	28 ± 2	1030 ± 82	1180 ± 106	6.08 ± 0.49
Ruri15	68 ± 5	426 ± 34	17 ± 1	511 ± 41	1342 ± 121	6.92 ± 0.55
Average	111 ± 09	1564 ± 125	38 ± 3	1713 ± 137	733 ± 66	3.78 ± 0.30

**Table 5:** Comparison of the average activity concentrations of^226^Ra, ^232^Th, and ^40^K, Ra_eq_, and AEDR in Homa, Ruri, and other HBRA regions in Kenya and others around the world.

Region	Country	Activity concentration (Bq/kg) ^226^Ra			Ra_eq_ (total) (Bq/kg) ^232^Th	AEDR (mSv/y) ^40^K	Reference
Homa	Kenya	129	399	894	767	1.7	This work
Ruri	Kenya	111	1094	489	1713	3.9	This work
Mrima	Kenya	134	431	249	—	1.8	[8]
Ramsar	Iran	179	29	202	144	—	[11]
Nile Delta	Egypt	107	201	116	404.8	—	[20]
Kanyakumari	India	31	206	1590	437	0.9	[21]

## Data Availability

The data used to support the findings of this study are available from the corresponding author upon request.
